# Boxes fabricated from plate-stabilized liquid marbles†

**DOI:** 10.1039/d1ma00398d

**Published:** 2021-06-11

**Authors:** Junya Fujiwara, Ai Yokoyama, Musashi Seike, Nicolas Vogel, Marcel Rey, Keigo Oyama, Tomoyasu Hirai, Yoshinobu Nakamura, Syuji Fujii

**Affiliations:** Division of Applied Chemistry, Graduate School of Engineering Osaka Institute of Technology, 5-16-1, Omiya, Asahi-ku Osaka 535-8585 Japan; Department of Applied Chemistry, Faculty of Engineering Osaka Institute of Technology, 5-16-1 Omiya, Asahi-ku Osaka 535-8585 Japan syuji.fujii@oit.ac.jp; Institute of Particle Technology, Friedrich–Alexander University Erlangen–Nürnberg Cauerstrasse 4 Erlangen 91058 Germany; Department of Physics and Astronomy, The University of Edinburgh, Peter Guthrie Tait Road Edinburgh EH9 3FD UK; Nanomaterials Microdevices Research Center, Osaka Institute of Technology, 5-16-1 Omiya, Asahi-ku Osaka 535-8585 Japan

## Abstract

Polyhedral liquid marbles were fabricated using hydrophobic polymer plates in the shape of a circle, a heart and a star as a stabilizer and water as an inner liquid phase. Boxes could be fabricated by the evaporation of the inner water from the liquid marbles. The fabrication efficiency and stability of these boxes as a function of the plate shape were investigated. Functional materials such as polymers and colloidal particles were successfully introduced into the boxes.

## Abstract 2

Boxes containing functional materials are fabricated from plate-stabilized liquid marbles *via* the evaporation of the inner liquid.

## Graphical abstract

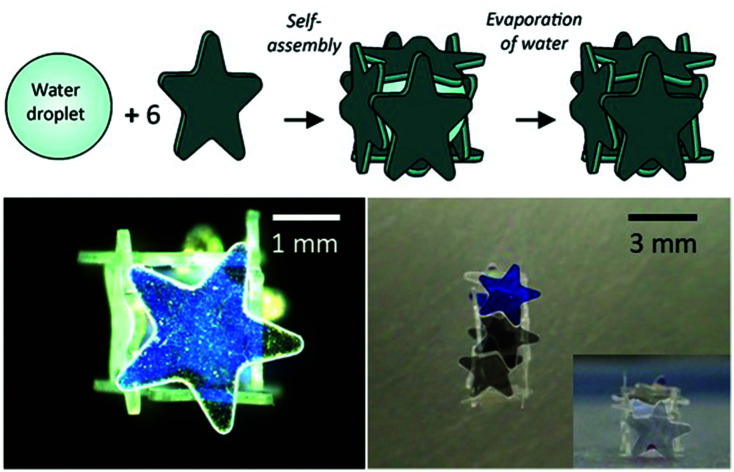

Particulate hollow materials have found applications in a variety of academic and industrial fields, including coatings, inks, drug delivery systems, catalysts and cosmetics, due to their excellent characteristics of low density, light scattering and capability of loading/release of materials.^1–6^ Among the particulate hollow materials, cubic/rectangular hollow materials, *i.e.* box materials, have started to receive considerable attention due to their unique morphology and light scattering, electrical and thermal properties and have been used as fillers for anti-slipping materials and light-emitting diode devices, sensors, electrodes for batteries and thermal insulators.^7–12^ There are mainly two fabrication methods toward box materials: (1) folding multiple two-dimensional molecularly designed plates (DNA origami domains) with interconnecting strands^7,8^ and (2) coating of cubic particles with shell materials, followed by the extraction of core cubic components.^9–12^ These fabrication methods are complex and multi-step processes, which are time and energy consuming and often require harsh chemicals. Therefore, there is a need for facile, energy-saving and environmentally acceptable methodologies.

Liquid droplets covered by hydrophobic solid particles adsorbed at the liquid–gas interface, which behave as non-wetting soft objects, are called liquid marbles (LMs).^13–17^ An LM is a unique liquid-in-gas soft dispersed system, which can be stabilized only by solid particles and not by molecular surfactants. In this respect, LMs are strikingly different from emulsions and foams, which can be stabilized by solid particles as well as molecular surfactants. Due to their ability to encapsulate functional moieties, LMs have been proposed in wide range of research areas, such as carriers for materials,^18,19^ microreactors,^20–25^ health care products,^26,27^ sensors,^28–31^ accelerometers,^32^ cell models^33^ and pressure-sensitive adhesives.^34^ To date, (near) spherical particles and their flocs have been generally used as the stabilizer and studies on LMs stabilized with well-defined non-spherical stabilizers monodispersed in shape and size have been limited. Recently, hydrophobic, monodispersed millimeter-sized polymer platelets were used as an LM stabilizer to stabilize polyhedral LMs.^35–37^ Taking into consideration the various applications of LMs, an investigation of the relationship between the shape of the stabilizer and the formability, stability and structure of LMs is crucial.

In this communication, the effects of plate shape on the formation, structure and stability against drying of LMs were investigated. Furthermore, we proposed the fabrication method of box materials from polyhedral LMs simply by the removal of the inner water *via* evaporation (Fig. 1a).

**Fig. 1:**
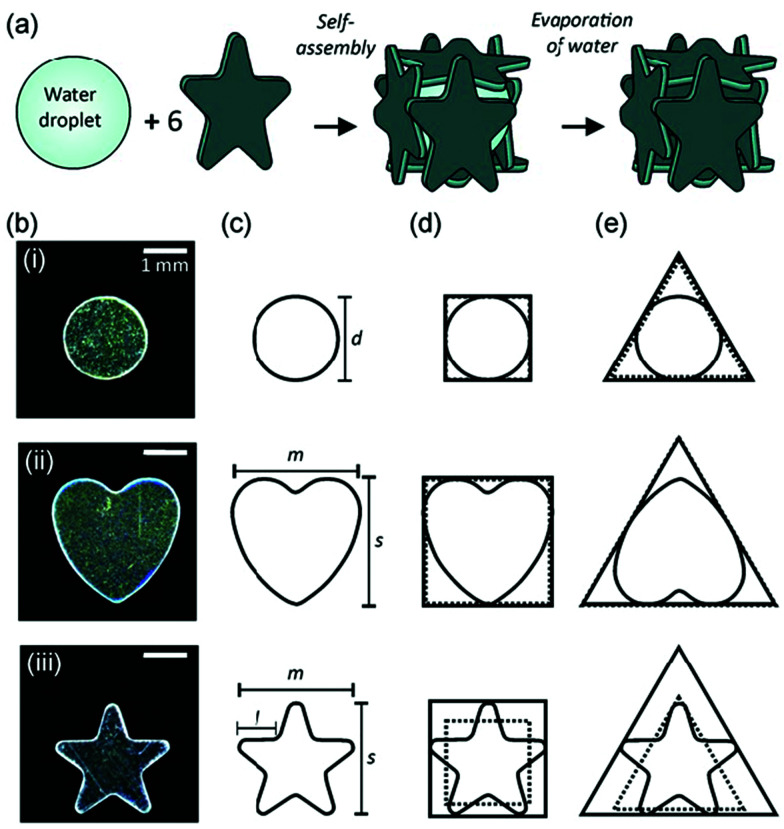
(a) Schematics illustrating fabrication of boxes from cube-shaped liquid marbles *via* the evaporation of the inner water. (b) Optical photographs and (c) schematics of PET plates used in this study: (i) circular-, (ii) heart- and (iii) star-shaped plates after hydrophobization. (d) Theoretical square tiles for the formation of a cubic box and (e) triangular faces for the creation of a tetrahedron assuming a tight fit (full line) and experimentally measured tile size (dotted line).

Poly(ethylene terephthalate) (PET) plates with circular, heart, and star shapes (A-17-P12 H-070, Daiso Industries Co., Ltd) were used as the LM stabilizer. All plates were monodispersed in shape and size, as confirmed by using optical and scanning electron microscopy (SEM) studies (Fig. 1b, c and Fig. S1, ESI†). The circular-shaped plates had a diameter (*d*) of 2.03 ± 0.04 mm and a thickness (*t*) of 154 ± 46 μm. The heart-shaped plates had a major axis (*m*) of 3.03 ± 0.02 mm, a side length (*s*) of 3.02 ± 0.02 mm and a thickness (*t*) of 91 ± 18 μm. The star-shaped plates had a major axis (*m*) of 2.88 ± 0.09 mm, a side length (*s*) of 2.75 ± 0.08 mm, a length of one side (*l*) of 0.88 ± 0.07 mm and a thickness (*t*) of 86 ± 24 μm. The original PET plates had relatively hydrophilic surfaces and water tended to wet the plates to form a plate–water aggregate rather than the formation of stable LMs. Therefore, hydrophobization of the plates was conducted using 1*H*,1*H*,2*H*,2*H*-perfluorodecyltrichlorosilane (ESI†). The static contact angle of a 5 μL water droplet on the fluorinated silane coupling agent-treated PET plates was 110° ± 5°, suggesting a successful surface modification.

If the water droplet was deposited and gently rolled on the dried hydrophobic plate bed, the plates autonomously adsorbed onto the droplet surface to form LMs for all different plate shapes. The LMs had hydrophobic and non-wetting characteristics and could be transferred from the plate bed to solid substrates, including hydrophilic glass slides or paper. The size of the LMs could be controlled in a range between *ca.* 3 mm and 10 mm simply by tuning the volume of the inner water droplet. Fig. 2 shows stereomicroscopy images of the LMs, which were prepared using circular-, heart-, and star-shaped PET plates with various water droplet volumes (10 μL, 15 μL, 20 μL, 50 μL, 100 μL and 200 μL), placed on a hydrophilic glass substrate. In all plate systems, polyhedral LMs were formed and an increase of water volume, allowing a larger number of plates to adsorb onto the droplet, increased sphericity. The LMs were stabilized with plate monolayers rather than multilayers, indicating that the gravitational force outcompeted plate–plate interactions and thus removed plates not in direct contact with the liquid.^38^ Due to the gravity effect, the LM shape started to deviate largely from (near) spherical to oblate at and above 50 μL (note that the capillary length of water is 2.7 mm).

**Fig. 2:**
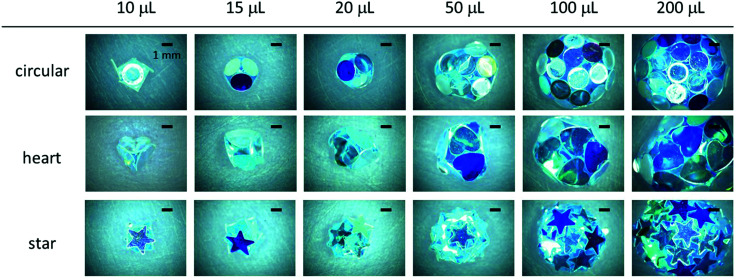
Liquid marbles (LMs) stabilized with hydrophobic PET plates with circular, heart and star shapes. The inner water volumes were varied from 10 μL to 200 μL.

In the case of circular-shaped plates, one large face of the plate was fully attached to the droplet surface, when the size of the droplet was adequately larger (here, >100 μL) than that of the plate. The plates formed a hexagonal two-dimensional crystal-like structure on the LM surface (Fig. 2 and Fig. S2, ESI†). When the sizes of the droplets were comparable to those of the plates (approximately 10 μL), parts of the plates, which could not bend by capillary forces, started to stick out of the surface of the LM into the air phase. For heart- and star-shaped plates, parts of the plates were stuck out of the surfaces of the LMs in a similar manner, and some plates partially overlapped with each other on the surface of the LMs independent of droplet size (Fig. 2 and Fig. S2, ESI†).

The position of the plate at the air–water interface was investigated using SEM microscopy of the LMs after ethyl 2-cyanoacrylate vapor treatment to solidify the liquid interface and therefore fix the position of the plates *via* anionic polymerization.^39^ These observations revealed that the plates were adsorbed as monolayers on the LMs (50 μL) (ESI† and Fig. S3). The air–water–plate contact lines were mainly pinned at the inner edge of the plates and one large face of the plate contacted with the water phase, as previously observed.^35,36^ The contact lines sometimes crossed to the side wall of the plates. The reason is unclear at this stage, but possible reasons include inhomogeneities in surface chemistries at the sides of the plates or the stress-induced release of the pinning of the wetting contact lines during fabrication.

Interestingly, LMs with notable polyhedral shapes (*e.g.* tetrahedron and cube) could be fabricated if the size ratio between the plate and the droplet was optimized (Fig. 3). There have been reports on a similar, but different, phenomenon known as capillary origami, where a highly elastic sheet wraps the droplet.^40,41^ In contrast, the PET plates used in this study are rigid and do not bend by capillary forces. Digital microscopy studies confirmed that the shape of the internal water in the liquid marble accorded with that of the liquid marble: *e.g.* the water inside of the cube-shaped liquid marble had a cube shape. (Fig. S4, ESI†). We first calculated the ideal water volume by tightly fitting the square and triangular faces around different plates (Fig. 1d, e, full line) and compared them to the measured volumes (Table 1 and ESI†). While the calculated and measured volumes are in good agreement for circular- and heart-shaped plates, the measured volume for the star-shaped plates is significantly lower compared to the calculations. This discrepancy arises because the tips of the stars stick out of the box (Fig. 2 and 3) and thus a lower water volume is required. We therefore calculated the actual face sizes from the measured volumes, which are shown in Fig. 1d and e as dotted lines.

**Fig. 3:**
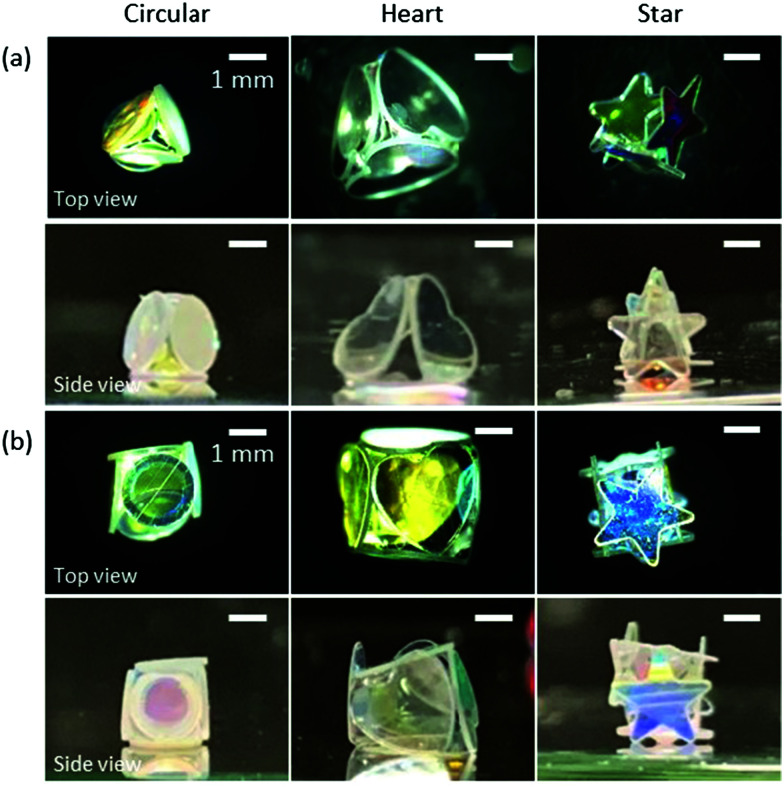
(a) Tetrahedron- and (b) cube-shaped LMs stabilized with hydrophobic PET plates with circular, heart and star shapes.

**Table 1:** Calculated and measured droplet volumes to obtain cube- or tetrahedron-shaped LMs stabilized with circular-, heart- or star-shaped plates

	Cube		Tetrahedron	
	Calculated volume [μL]	Measured volume [μL]	Calculated volume [μL]	Measured volume [μL]
Circular	8.4	8.0	5.1	4.0
Heart	27.8	25.0	11.5	12.0
Star	23.9	9.0	13.5	4.0

Next, we investigated the morphological changes of polyhedral LMs with tetrahedral and cubic shapes during the evaporation of the inner liquid (Fig. S5, ESI†). It should be noted that some polyhedral LMs kept their original three-dimensional shapes to form the box structure even after the complete evaporation of water (Fig. 4a and b). Digital microscopy studies indicated that an air void was formed in the water phase and grew over time during evaporation (Fig. S4, ESI†). This was completely different from conventional LMs stabilized with nano- and micrometer-sized solid particles, which show buckling and wrinkles on their surfaces without the formation of air voids in the water phase during/after the evaporation of inner liquids.^42–45^ The plate shape had a strong effect on the stability of the tetrahedral and cubic LMs against the evaporation of the inner water to form boxes. Regarding tetrahedral LMs, 0%, 50%, and 70% LMs could maintain their original three-dimensional structures for circular-, heart- and star-shaped plate systems (Fig. S6a, ESI†). The survival percentages for cubic LMs (the yield of empty boxes) were 10%, 30% and 90% (Fig. S6b, ESI†). These results indicated that the stability of the LMs against drying increased with the degree of overlap of the plates as the mechanical stability of the LMs increases with an increase of the degrees of steric hindrance and interlocking among the plates.

**Fig. 4:**
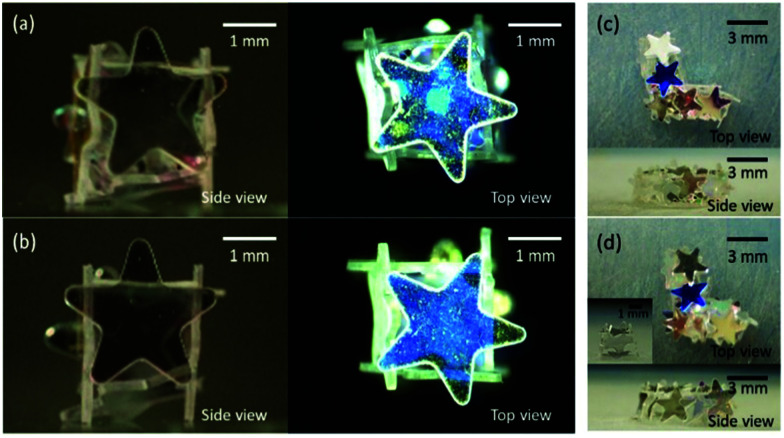
(a and b) Cube-shaped and (c and d) “L”-shaped LMs stabilized with hydrophobic star-shaped PET plates (a and c) before and (b and d) after the removal of the inner water *via* evaporation. Inset of (d) shows the cubic box after the removal of one plate.

The stability against impact was correlated with the mechanical integrity of the LMs estimated based on the minimum potential energy above which their shapes were destroyed (*E*_m_) utilizing a customized drop stability evaluation apparatus^38,46,47^ (Table S1 and Fig. S7, ESI†). The potential energy (*E*) was calculated based on eqn (1).

1 *E* = *mgh*

Here, *m* is the weight of an LM, *g* is the gravitational acceleration (9.81 m s^−2^) and *h* is the drop height. We counted LMs that kept their initial shape as “survived” and those disintegrated as “destroyed”. The *E* values affect the mechanical stability of the LMs against drop impact: the larger *E* values should cause deformations of the LMs to a larger extent, leading to easier destruction. The *E*_m_ values were measured to be 89 × 10^−7^, 78 × 10^−7^ and 131 × 10^−7^ J for the LMs stabilized with circular-, heart- and star-shaped plates, and the star-shaped plate system with the highest interlocking showed the highest stability.

Shape-designability is one of the advantages of the LMs stabilized with plates.^35,36^ It is worth noting that “I”-shaped LMs could be fabricated by merging multiple LMs (Fig. S8, ESI†). Furthermore, the shape of the LMs could be plastically rearranged to an “L”-shape by the application of an external force using a pipette tip, and the LMs behaved as liquid plasticines^48,49^ (Fig. 4c and Fig. S8, ESI†). These complex shapes indicate a strikingly different behavior compared to bare water droplets, which have shapes with lower surface areas to minimize the interfacial energy. The LMs had an inelastic character which shows plastic deformation due to the steric jamming of plates^50–52^ and the letter shaped-LMs did not experience a Rayleigh–Plateau capillary instability^53^ (“I”-shaped LMs with an aspect ratio of > 15 did not break up into smaller LMs). Similar kinetically trapped shapes realized due to the jamming of solid particles have been also reported for other solid particle-stabilized soft dispersed systems including armored bubbles and droplets^54–56^ as well as bicontinuous interfacially jammed emulsion gels.^57^

The structural stability of the “I”- and “L”-shaped LMs against drying was then studied (Fig. 4c, d and Fig. S8, ESI†). LMs stabilized with circular- and heart-shaped plates completely broke down and lost their three-dimensional structure after drying, whereas those stabilized with star-shaped plates kept their three-dimensional structure (three-dimensional structure retention percentage, ∼90%) to form “I”- and “L”-shaped boxes. These results again indicate that the star-shaped plates with higher degrees of steric hindrance and interlocking among the plates showed a higher mechanical stability of the LMs.

The mechanical stability of the box materials could be further improved by the addition of poly(*N*-vinylpyrrolidone) (PNVP, 1.0 wt%) to the encapsulated liquid, which forms a film inside of the LM and fully prevents the boxes stabilized by star-shaped plates from disintegrating after the evaporation of the inner liquid (Fig. S9, ESI†). Furthermore, functional particles such as magneto-responsive carbonyl iron particles (number average diameter, 2.8 ± 0.6 μm; 10 wt%) could be dispersed in the inner liquid containing PNVP and allow the formation of the box which can be magnetically manipulated (Fig. 5a and Fig. S10, ESI†). Once a magnetic bar on the top of a glass slide approached the box to a distance of approximately 2 cm, the box was vertically lifted and attached to the glass. Subsequently, the box could be transported at will and released at a desired location by removing the magnetic bar from the glass slide (Fig. 5b). Furthermore, it was demonstrated that the box showed circular motions on the magnetic stirrer (Fig. 5c and ESI†).

**Fig. 5:**
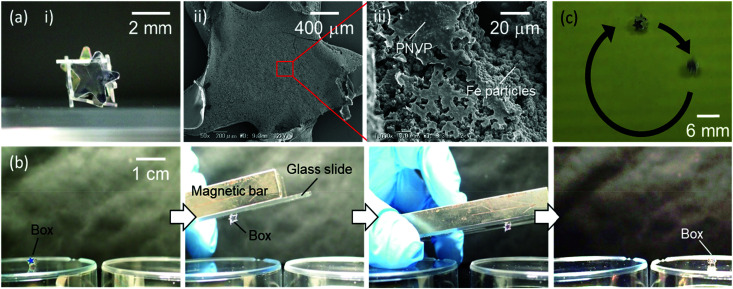
(a) Cube-shaped LMs, containing the aqueous solution of poly(*N*-vinylpyrrolidone) (PNVP, 1 wt%) and carbonyl iron particles (10 wt%), stabilized with hydrophobic star-shaped PET plates after the removal of the inner water *via* evaporation. (i) Photographs and (ii and iii) SEM images of the PET plate-stabilized LMs. SEM images show the inner side of the PET plate attached to the bottom part of the LM. (b) The transfer of the cube-shaped box made from the LM containing PNVP and carbonyl iron particles using a magnetic bar. (c) The rotational motion of the box driven using a magnetic stirrer.

In conclusion, LMs can be successfully fabricated using hydrophobic PET plates with circular, heart and star shapes. The shape of the LMs depends on the number of plates adsorbed onto the droplet surface and the size ratio of the plate and droplet diameter. When the plate size is smaller compared to the droplet size, near-spherical and oblate-shaped LMs were prepared. When the size ratio of the plate and the droplet is comparable, tetrahedral and cubic polyhedral LMs could be prepared. The structural stability of the resulting LMs depends on the shape of the plates and increases with an increasing overlap and interlocking of the stabilizing plates. The box materials could be fabricated simply by the evaporation of inner liquids from the LMs. The use of the plates as an LM stabilizer enables the shape design of droplets/boxes and proposes the methodology for the generation of plate-based buildings with three-dimensional architectural structures. Furthermore, functional materials (*e.g.* polymer and colloidal particles) can be easily introduced into the box without leakage to provide external actuation and locomotion *via* magnetic properties.

## Author contributions

Junya Fujiwara contributed to the conceptualization, methodology, and investigation. Ai Yokoyama contributed to the conceptualization, methodology, and investigation. Musashi Seike contributed to the methodology and investigation. Nicolas Vogel contributed to the writing – review and editing and funding acquisition. Marcel Rey contributed to the methodology, writing – review and editing, and funding acquisition. Keigo Oyama contributed to the methodology and investigation. Tomoyasu Hirai contributed to the methodology and investigation. Yoshinobu Nakamura contributed to the methodology and investigation. Syuji Fujii contributed to the conceptualization, methodology, writing – original draft, writing – review and editing, supervision, project administration, and funding acquisition.

## Conflicts of interest

There are no conflicts to declare.

## Supplementary Material

MA-002-D1MA00398D-s001
