# Supplementary material for: Boxes fabricated from plate-stabilized liquid marbles
Source: Mater Adv. 2021 Jun 11;2(14):4604–9. doi: 10.1039/d1ma00398d (PMC8290327; doi:10.1039/d1ma00398d)
Supplement: MA-002-D1MA00398D-s001 [file MA-002-D1MA00398D-s001.pdf]

Electronic Supplementary Material (ESI) for Materials Advances.
This journal is © The Royal Society of Chemistry 2021

Electronic Supplementary Information

Materials Advances manuscript

'Box fabricated from plate-stabilized liquid marble'

by J. Fujiwara *et al.*

Corresponding author:

Prof. Syuji Fujii; syuji.fujii@oit.ac.jp

Materials

Experiments were carried out at room temperature. The following chemicals were used: 1*H*,1*H*,2*H*,2*H*-perfluorodecyltrichlorosilane (PFDTS, 96%, TCI), *n*-hexane (96%, Wako), ethanol (95%, Sigma-Aldrich), ethyl 2-cyanoacrylate (Aron Alpha Extra Sokkotayoto, Toagosei Co., Ltd.), poly(*N*-vinylpyrrolidone) (PNVP; nominal molecular weight = 360 kD), and carbonyl iron particles (number-average diameter, 2.8±0.6 μm; Carbonyl Iron Powder CS, BASF). Purified and deionized water from Advantec MFS RFD240NA (GA25A-0715) was used for all experiments. Poly(ethylene terephthalate) (PET) plates in shapes of circle, heart and star (A-17-P12 H-070) were purchased from Daiso Industries Co., Ltd. Surface areas of one large plate face were calculated to be 3.52±0.21, 7.16±0.14 and 4.00±0.09 mm^2 for the circular-, heart- and star-shaped plates, respectively.

Hydrophobization of the plates

The pristine plates (1-5 g) were washed repeatedly with ethanol (50-100 mL) until the supernatant became clear (3-4 times). The washed plates were dried in air for 24 h and then in vacuum overnight and then modified with PFDTS. The dried plates (1-5 g) were suspended in a *n*-hexane (40 mL) solution of 40 μL PFDTS and the mixture was shaken for approximately 30 s. During the reaction time of 30 min, the sedimented plates were redispersed by hand shaking every 5 min to ensure a homogenous hydrophobization of the plates. Subsequently, the surface-modified plates were rinsed 3 times with *n*-hexane, and were dried in air for 24 h and then in vacuum overnight.

Preparation of liquid marbles

The liquid marbles (LMs) were prepared by simply rolling water droplets over a plate bed on surface-roughened poly(chlorotrifluoro ethylene) (PCTFE) petri dish. Afterwards, the prepared LMs may be transferred to other substrates like glass slides using a (plastic) spoon.

Trapping of plates at surfaces of LMs

LM was allowed to stand for 30 minutes in a glass container filled with superglue (whose main component is ethyl 2-cyanoacrylate; ECA) vapor to cause anionic polymerization at the air-water interface on the surface of LM, as described in detail elsewhere [1, 2]. To make the ECA vapor, the ECA was heated about 50˚C.

Adsorption energy of the plate at air-water interface

When drying the near-spherical LMs with large water volumes, buckling was observed on the surfaces of LMs in all plate shape systems. Such buckling should be caused by the surface jamming effect of the plates at the LM surfaces. The surface jamming could occur due to high adsorption energy (ΔG) of the plates at interface, which is defined as the difference in free energy between a system where one large face of the plate contacts with water and the other faces contacts with air compared to the state where all faces of the plate are exposed to air plus a free air-water interface. ΔG values of the plates with circular, heart, and star shapes were estimated to be 1.7×10^{-8} J, 3.4×10^{-8} J, and 1.9×10^{-8} J, using $\gamma_{AS} = 5$ mN m^{-1} [3], $\gamma_{WS} = 30$ mN m^{-1}, and $\gamma_{AW} = 72$ mN m^{-1} [4], which are air-plate, water-plate, and air-water interfacial energies, respectively (γ_{WS} was determined using Young's equation with a water contact angle of 110˚). These ΔG values are more than three orders of magnitude higher than those of micrometer-sized spherical particles adsorbed at the air-water and oil-water interfaces [5, 6], indicating that the adsorption is irreversible and the plates did not flip off or detach during evaporation of inner water at room temperature. Therefore, the volume decreases of LMs during the evaporation of water were compensated by deformation (generation of wrinkles) with keeping the total surface areas almost constant.

Observation with a stereoscopic microscope

The surface of the LM was observed from the top using a stereo microscope (Shodensha Co., Ltd., TG300PC3 fitted with GR 300 BCM 2 camera). From the microscopy images, the position of each plate was extracted using a custom-written Matlab software

based on the publicly available particle tracking algorithm by Crocker and Grier [7]. The nearest neighbors of each plate were found by Voronoi tessellation. For the visualization, Voronoi cells of plates with 6 neighbors are shown in green, plates with 5 and 7 neighbors are shown in blue and red, respectively. The 2D hexagonal order parameter Ψ_6 was calculated by using the formula (S1):

$$\Psi_6 = \langle \frac{1}{N_b} \rangle \sum_{j=1}^{N_b} \exp(i n \theta_j) \quad \text{(S1)}$$

Here, N_b is the number of nearest neighbors, θ_j is the bond angle between the plate and its nearest neighbors and n is set to 6. We used the standard error as error bar. A Ψ_6 parameter of 1 corresponds to perfect hexagonal order.

Ideal volume to obtain cubes and tetrahedrons

For the calculations of the ideal volumes to obtain cubes and tetrahedrons, the square and triangular faces were fitted tightly around the plates with circular, heart, and star shapes. The volume of the cube V_c was calculated using formula (S2),

$$V_c = a^3 \quad \text{(S2)}$$

where a is the length of the square face, corresponding to diameter d for circular-shaped plates or to the major axis m for heart- and start-shaped plates. Similarly, the volume of the tetrahedrons V_t was calculated using formula (S3),

$$V_t = c^3\, 2^{1/2} / 12 \quad \text{(S3)}$$

where c is the edge length of the triangular face. The measured sizes of the faces were obtained by solving for a and c respectively with the known volumes from **Table 1**.

Mechanical integrity of LMs

The mechanical integrity of LMs was investigated using a customized droplet test apparatus **(Figure S5)** [8]. LMs stabilized with different plate systems were placed on a cube-shaped silicone substrate (composition KE-26/CAT-RM/CAT-24 in mass ratio 100:1:4.5, Wacker Asahikasei Silicone Co., Tokyo, Japan) with dimensions of 30 × 27 × 10 mm^3. The silicone substrate carrying the LM was fixed using a clamp at various

heights above a flat surface (lab bench) and then dropped by loosening the clamp. The vertical distances between the bottom of the silicone substrate and the bottom surface were varied between 5 and 130 mm. The outcomes of this test were classified as "survived", if the LM keeps cubic shape and as "destroyed", if the cubic shape of the LM was destroyed.

Manipulation of magneto-responsive LMs.

A magnetic bar (150 mm×10 mm×30 mm, 533 mT) was obtained from NeoMag Co. Ltd and was used for manipulation of the LM containing carbonyl iron particles and PNVP. A magnetic stirrer (RS-1DN, AS ONE Co.) was utilized to induce rotation motion of the LM at 150 rpm.

1. N. Vogel, J. Ally, K. Bley, M. Kappl, K. Landfester and C. K. Weiss, *Nanoscale*, 2014, **6**, 6879-6885.
2. J. M. Chin, M. R. Reithofer, T. T. Tan, A. G. Menon, E. Y. Chen, C. A. Chow, A. T. Hor and J. Xu, *Chem. Commun.*, 2013, **49**, 493-495.
3. F. Geyer, Y. Asaumi, D. Vollmer, H. J. Butt, Y. Nakamura and S. Fujii, *Adv. Funct. Mater.*, 2019, **29**, 1808826.
4. N. B. Vargaftik, B. N. Volkov and L. D. Voljak, *J. Phys. Chem. Ref. Data*, 1983, **12**, 817-820.
5. S. Fujii, Y. Yokoyama, Y. Miyanari, T. Shiono, M. Ito, S. Yusa and Y. Nakamura, *Langmuir*, 2013, **29**, 5457-5465.
6. S. Fujii, Y. Yokoyama, S. Nakayama, M. Ito, S. I. Yusa and Y. Nakamura, *Langmuir*, 2018, **34**, 933-942.
7. J. C. Crocker and D. G. Grier, *Phys. Rev. Lett.*, 1996, **77**, 1897-1900.
8. Y. Nonomura, T. Tanaka and H. Mayama, *Langmuir*, 2016, **32**, 6328-6334.

Table S1. Characterization of the mechanical integrity of the LMs containing water stabilized with various-shaped PET plates using drop stability evaluation apparatus.

Circular PET plates [a)]

Height / mm		20	30	40	50	60	70	80	90	100
Potential energy / 10^{-7} J		22.15	33.22	44.30	55.37	66.44	77.52	88.59	99.67	110.74
Survived / numbers		3	3	3	2	1	2	0	0	0
Destroyed / numbers		0	0	0	1	2	1	3	3	3

Heart-shaped PET plates [b)]

Height / mm	5	10	20	23.8	27.2	30	40	50	60	70	80	90	100
Potential energy / 10^{-7} J	16.27	32.54	65.07	77.52	88.49	97.61	130.14	162.68	195.22	227.75	260.29	292.82	325.36
Survived / numbers	3	1	1	0	0	0	0	0	0	0	0	0	0
Destroyed / numbers	0	2	2	3	3	3	3	3	3	3	3	3	3

Star-shaped PET plates [c)]

Height / mm	20	30	40	50	60	70	80	90	100	110	120	130	140	150	160
Potential energy / 10^{-7} J	26.26	39.40	52.53	65.66	78.79	91.92	105.06	118.19	131.32	144.45	157.58	170.72	183.85	196.98	210.11
Survived / numbers	3	3	3	0 [d)]	1	1	1	1	0	0	0	0	0	0	0
Destroyed / numbers	0	0	0	3 [d)]	2	2	2	2	3	3	3	3	3	3	3

[a)] Mass of LMs stabilized with hydrophobic circular PET plates (Internal liquid: Water (8 μL)): 0.0113 g

[b)] Mass of LMs stabilized with hydrophobic heart-shaped PET plates (Internal liquid: Water (25 μL)): 0.0332 g

[c)] Mass of LMs stabilized with hydrophobic star-shaped PET plates (Internal liquid: Water (9 μL)): 0.0134 g

[d)] When the experiments were conducted 5 times, 2 LMs survived and 3 LMs were destroyed.

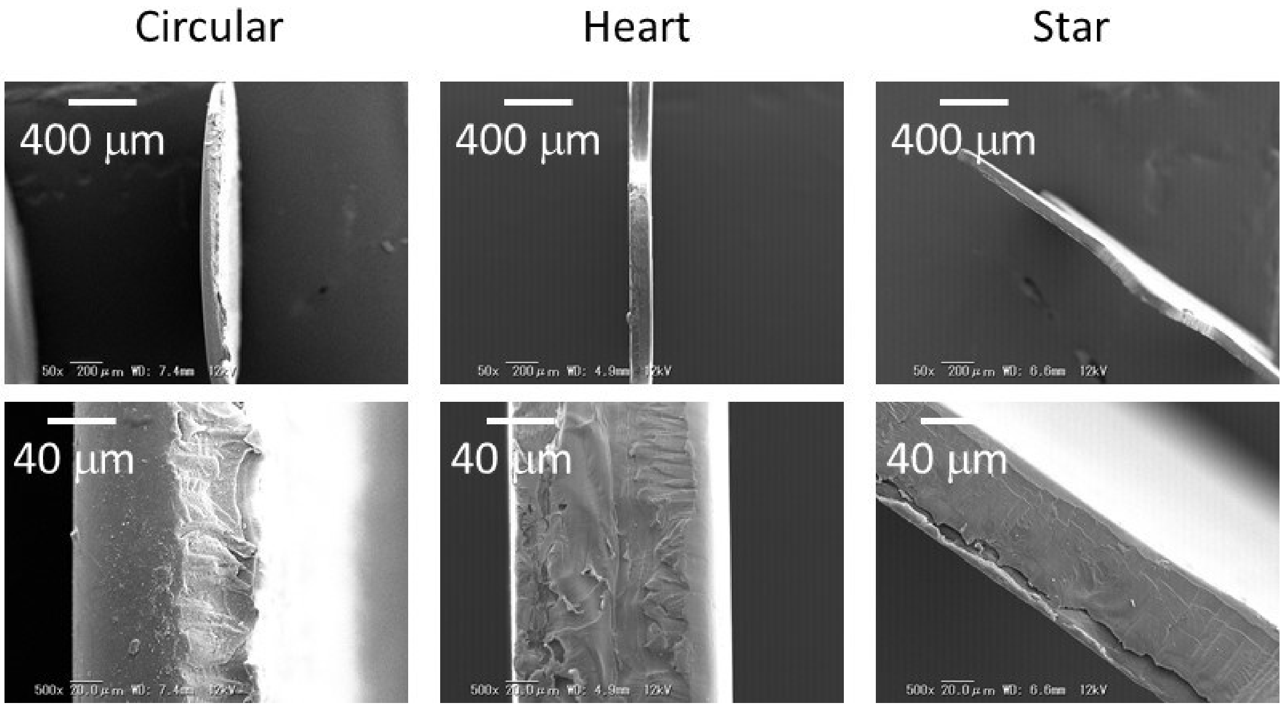

Figure S1. Cross-sectional SEM images of hydrophobic PET plates with shapes of circular, heart and star.

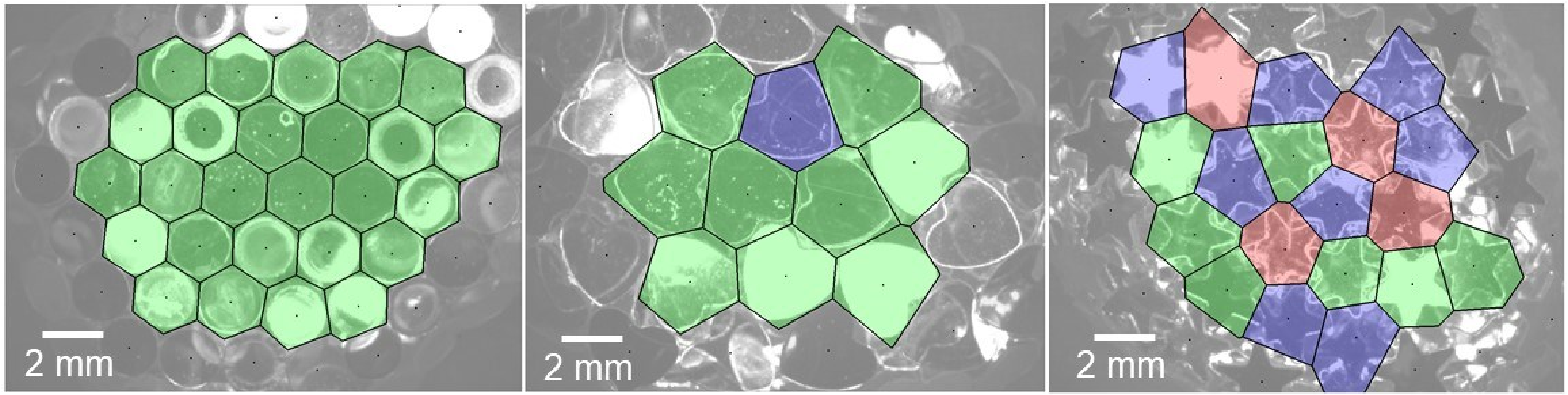

Figure S2. (a) Voronoi tessellation to extract order and arrangement. Plates with 6 neighbors are shown in green, plates with 5 and 7 neighbors are shown in blue and red, respectively.

The PSI6 value for the respective particles are 0.92±0.01 (circular), 0.55±0.04 (heart) and 0.62±0.03 (star). PSI6 value for hearts is lowest because they have an elongated shape, which prevents them from assembling into a hexagonal lattice. Both values (star and heart) are though very low compared to the circular plates.

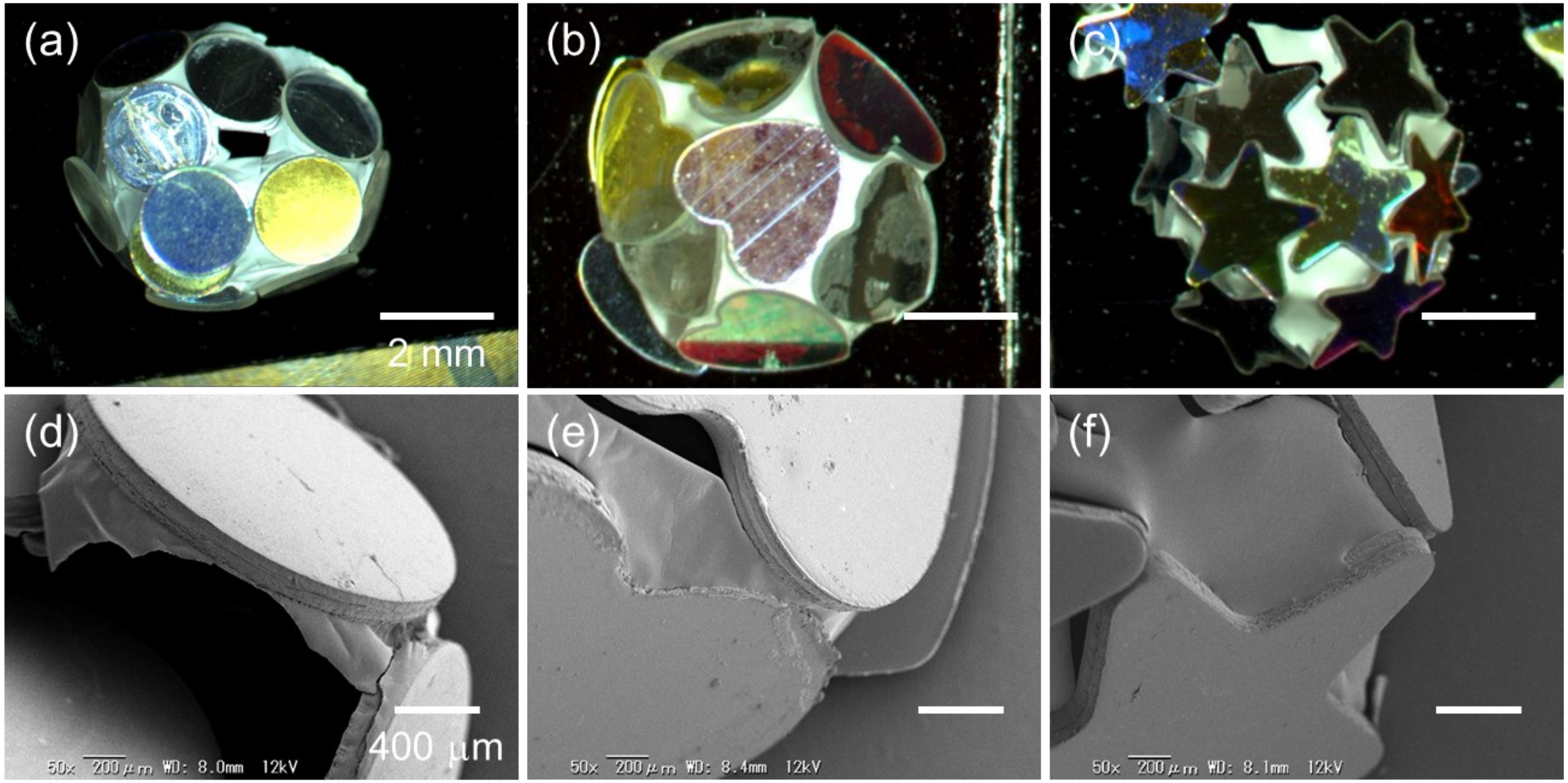

Figure S3. (a, b, c) Optical photographs and (d, e, f) SEM images of polyhedral LMs (50 μL) stabilized by PET plates with circular, heart, and star shapes after interfacial anionic polymerization of ethyl-2-cyanoacrylate, followed by evaporation of water.

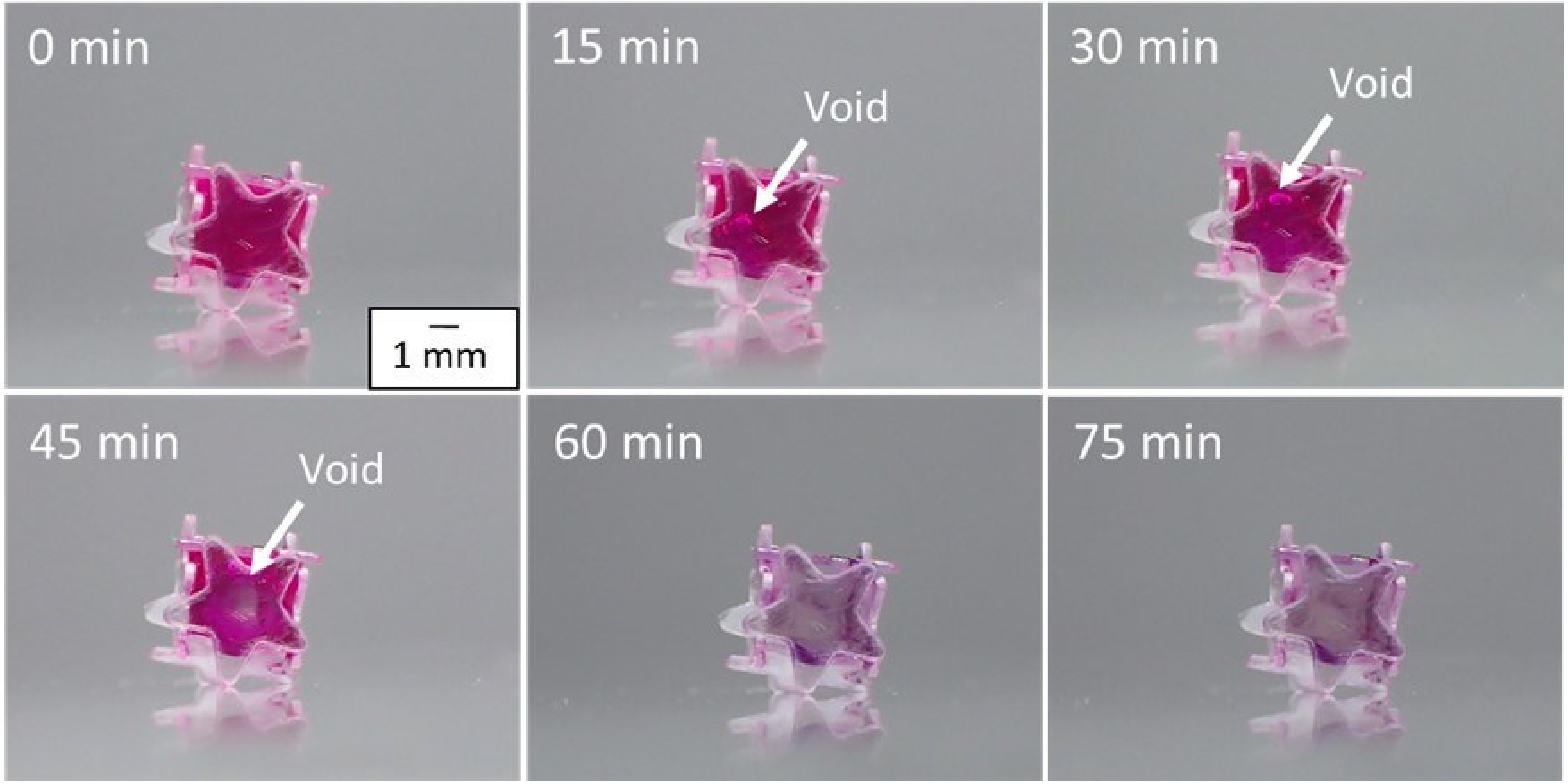

Figure S4. Drying process of cube-shaped LM (9 μL) stabilized with hydrophobic star-shaped PET plates (temperature, 24.9ºC; humidity, 44.3%). An arrow indicates air void generated in the water phase during evaporation of water. Internal liquid was dyed with Powder Sun Red®YM.

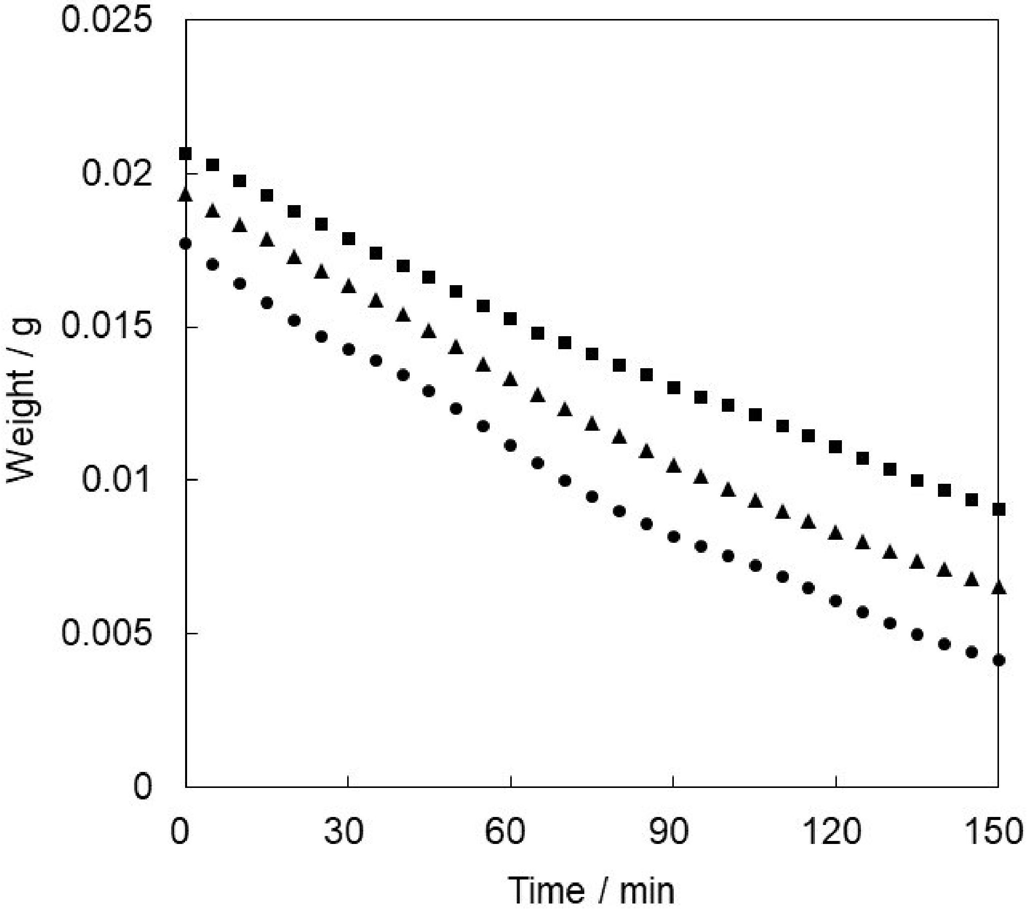

Figure S5. Weight of LMs (15 μL) stabilized with the hydrophobic PET plates with (●) circular, (■) heart and (▲) star shapes as a function of evaporation time. Evaporation rate: 5.4 mg/h, 4.6 mg/h and 5.2 mg/h for circular, heart and star systems.

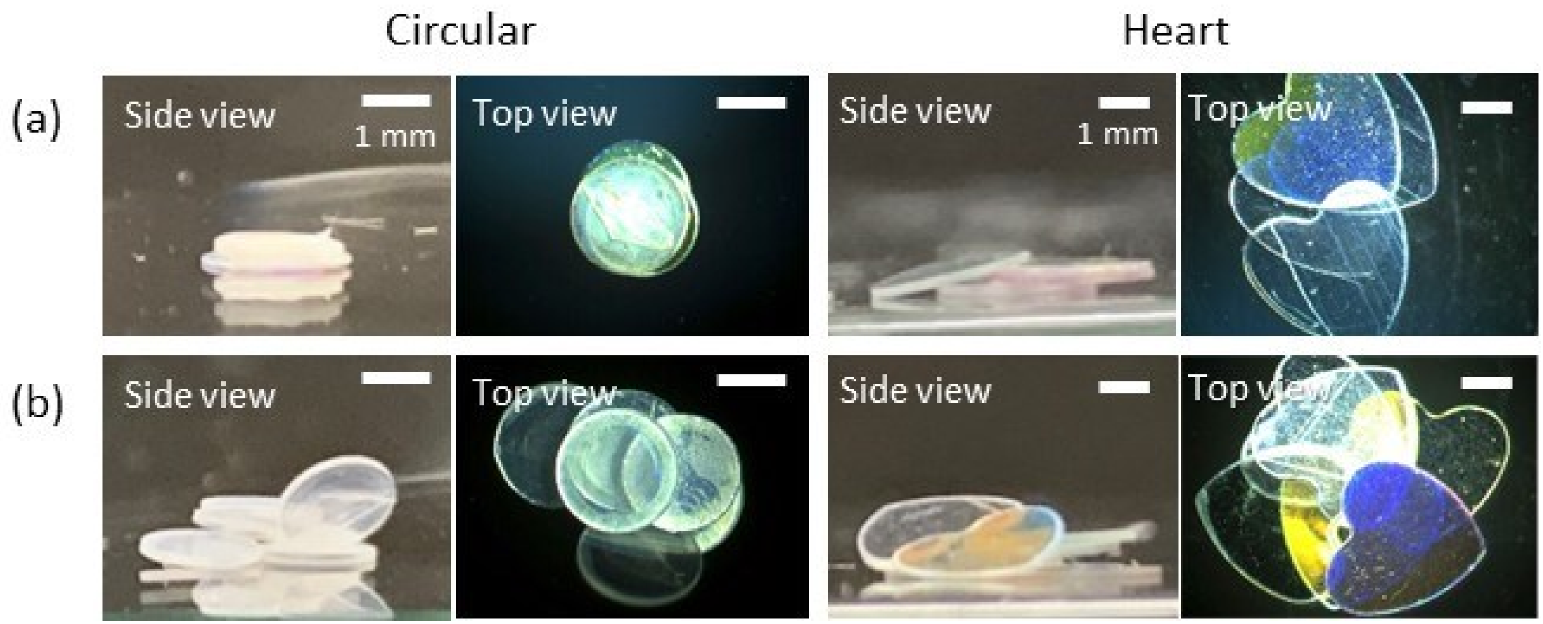

Figure S6. Optical photographs of (a) tetrahedron and (b) cube-shaped LMs stabilized with hydrophobic PET plates with shapes of circular and heart after removal of water via evaporation.

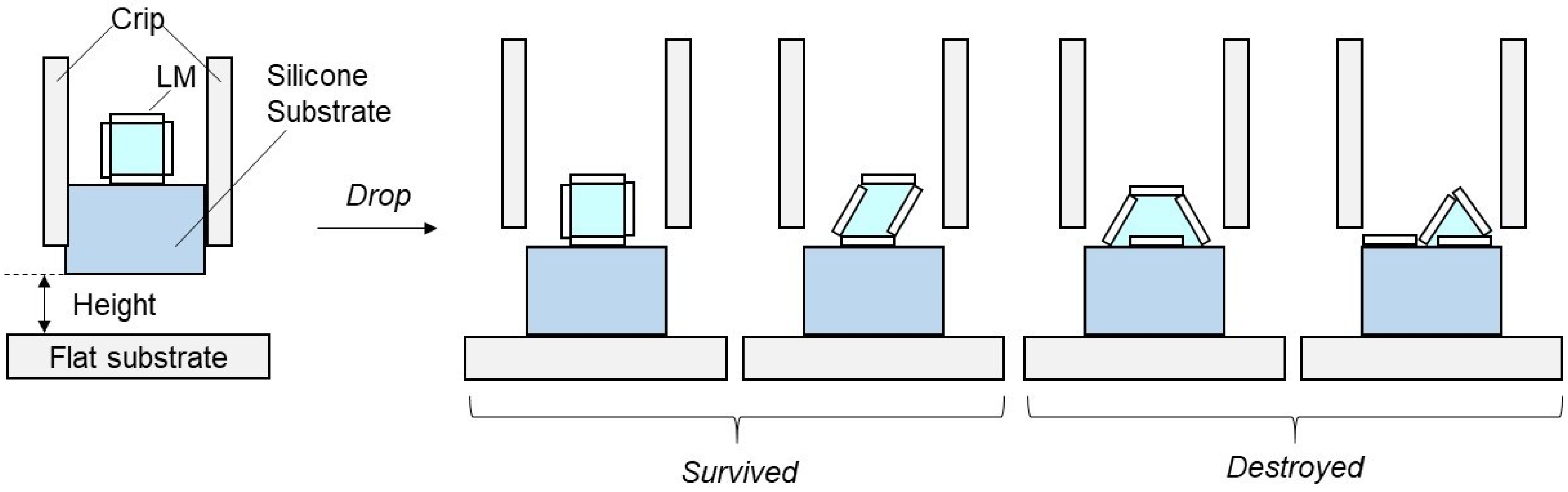

Figure S7. Home-made apparatus designed to evaluate the mechanical integrity of LMs. The vertical distances between the bottom of the silicone substrate and the flat substrate were varied between 5 and 160 mm. The outcomes were categorized into two states: survived and destroyed.

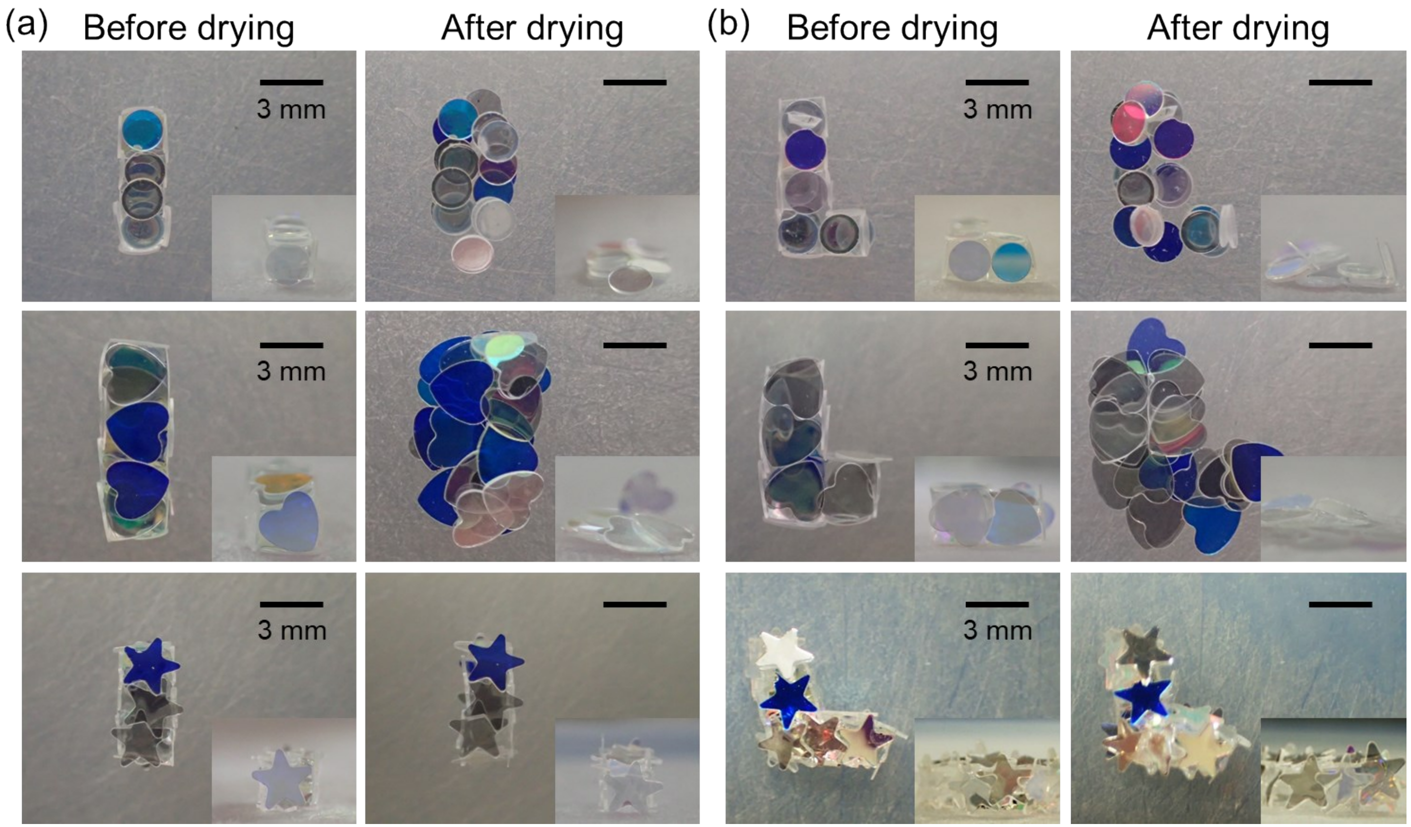

Figure S8. (a) "I"- and (b) "L"-shaped LMs stabilized with hydrophobic PET plates with shapes of circular, heart and star before and after removal of inner water via evaporation. Insets show side views of the LMs.

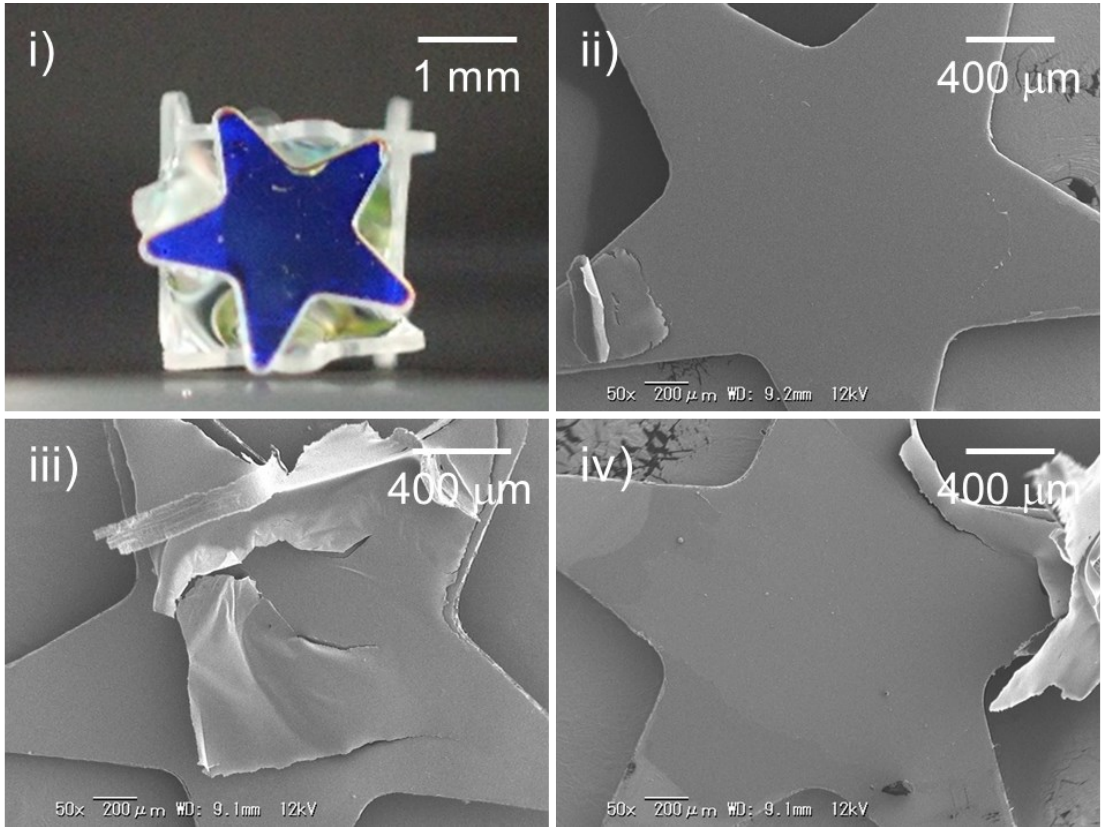

Figure S9. Cube-shaped LM, containing aqueous solution of poly(*N*-vinylpyrrolidone) (PNVP, 1 wt%), stabilized with hydrophobic star-shaped PET plates after removal of inner water via evaporation. i) Digital and ii-iv) SEM images of the PET plates stabilizing LMs. Inner sides of the PET plates attached to ii) top, iii) side and iv) bottom parts of the LMs.

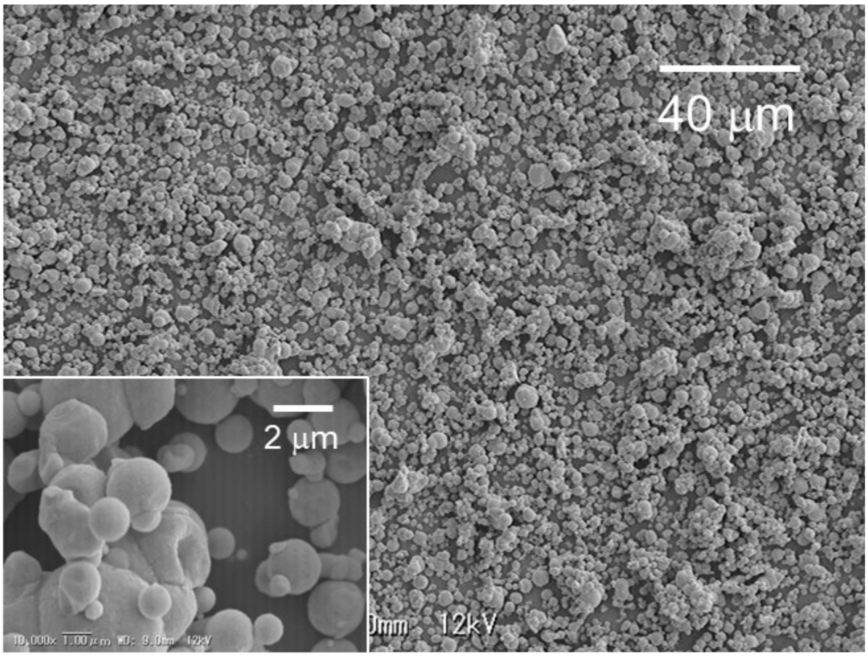

Figure S10. SEM images of carbonyl iron particles.
